# Coronary angiography was used to assess the effect of diabetes on off-pump coronary artery bypass graft patency

**DOI:** 10.1097/MD.0000000000039178

**Published:** 2024-08-02

**Authors:** Caiwu Zeng, Xiaomi Li, Ye Zhou, Nan Liu

**Affiliations:** a Center for Cardiac Intensive Care, Beijing Anzhen Hospital, Capital Medical University, Beijing, China.

**Keywords:** angiography, diabetes, mean graft flow, off-pump coronary artery bypass graft

## Abstract

This study aimed to examine the influence of diabetes on the left internal mammary artery (LIMA) and saphenous vein (SV) graft failure for 5-year follow-up. We enrolled 202 patients who underwent isolated off-pump coronary artery bypass grafting (CABG) surgery in 2014, angiographic follow-up occurred at 5 years after surgery. Angiographic outcomes in patients with or without diabetes were analyzed. Multivariate logistic regression analysis was used to identify independent predictors of graft dysfunction. A total of 66 (32.7%) patients had diabetes. Five-year rates of LIMA and SV graft failure were similar in patients with and without diabetes. In addition, in diabetics, the proportion of complete graft failure was significantly lower in the LIMA grafts (12/66, 18.2%) than in the SV grafts (57/133, 42.9%) (*P* = .001). In nondiabetic, the proportion of complete graft failure was also significantly lower in the LIMA grafts (28/136, 20.6%) than in the SV grafts (105/275, 38.2%) (*P* < .001). Multivariate logistic regression analysis showed that mean graft flow (MGF) was an independent predictor factor for LIMA (odds ratio = 1.186, 95% CI = 1.114–1.263, *P* < .001) and SV (odds ratio = 1.056, 95% CI = 1.035–1.077, *P* < .001) graft failure. Diabetes did not influence the patency of LIMA or SV grafts over a 5-year follow-up. LIMA grafts should be maximized in patients undergoing off-pump CABG surgery. Diabetes does not affect the patency of grafts CABG. Using angiography, our study proved that diabetes does not affect the patency of grafted vessels after CABG for 5 years.

## 1. Introduction

Patients with diabetes are not only prone to diffuse and rapidly progressive atherosclerosis^[1]^ but also have more severe coronary artery stenosis.^[2]^ It was reported that patients with diabetes have significantly lower survival after coronary artery bypass grafting (CABG).^[3]^ CABG has been regarded as the standard therapy in diabetic patients with multivessel coronary artery disease.^[4,5]^ When undergoing CABG, patients with diabetes have poorer clinical outcomes than patients without diabetes.^[6,7]^ However, there still exists controversy on bypass graft patency in patients with versus without diabetes.^[8–12]^ Therefore, in this study, we aim to elucidate the essential relationship between graft patency and diabetes in CABG patients.

## 2. Materials and methods

This study protocol conformed to the ethical guidelines of the 1975 Declaration of Helsinki as reflected in a priori approval by the human research committee of Beijing Anzhen Hospital, Capital Medical University. The written informed consent was acquired from each patient. The study was approved by the Institutional Ethics Committee of Beijing Anzhen Hospital, Capital Medical University.

From January 2014 to December 2014, a total of 311 patients underwent isolated off-pump CABG in Beijing Anzhen Hospital, Capital Medical University. The inclusion criteria for patients were stable angina, left ventricle ejection fraction of more than 50%, and left ventricular end-diastolic diameter of less than 60 mm. The exclusion criteria were emergency state, unstable angina, left ventricle ejection fraction less than 50%, left ventricular end-diastolic diameter more than 60 mm, comorbid with other heart diseases (valvular disease, congenital heart disease, dissection, etc.), and previous heart surgery.

### 2.1. Operative procedures

All procedures were performed through a median sternotomy, standard cannulation, and off-pump procedure stabilizers. The left internal mammary artery (LIMA) was harvested as pedicles and saphenous vein (SV) grafts were harvested by the open technique. End-to-side anastomoses were performed in a single continuous fashion with 6-0 for the proximal aortic connections during partial aortic clamping and with 7-0 for the terminal bypass. Side-to-side anti-parallel anastomoses were performed in a single continuous fashion with 7-0 for the sequential bypasses.

### 2.2. Post-operation and follow-up

**Figure 1.**
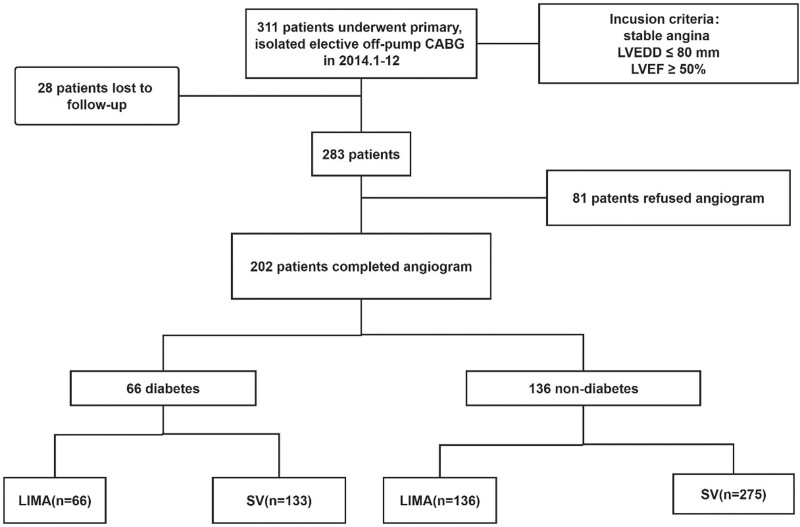
Flow diagram of the study population. CABG = coronary artery bypass grafting, LIMA = left internal mammary artery, LVEDD = left ventricular end-diastolic diameter, LVEF = left ventricle ejection fraction, SV = saphenous vein.

The definition of diabetes was based on medical history in the medical record. The treatment of diabetes was similar to the preoperative discretion of the treating physician. Most patients with diabetes were treated with insulin or with oral medication. In addition, other medical therapy included aspirin (100 mg/d orally) and atorvastatin calcium tablets (10 mg/d orally) in all cases, combined with clopidogrel (75 mg/d, orally) for 1 year. All other medications were prescribed as clinically indicated. Postoperative information was obtained by direct contact with the patients or through telephone conversations with patients or their families. Routine clinical assessment was performed each year. During 5-year follow-up, 28 patients lost to follow-up and 81 patients refused angiogram. The other 202 (65%) patients accepted coronary artery angiography and were further studied. According to the patient’s diabetes status, they were divided into 2 groups: diabetic patients (n = 66) and nondiabetic patients (n = 136) (Fig. 1).

### 2.3. Postoperative angiography

The angiographic recordings were made in the standard views and evaluated by 2 or more independent cardiologists. Each graft was assessed according to the Fitzgibbon classification.^[13]^ Each coronary anastomosis has been considered to be the distal end of a single bypass graft irrespective of the trunk configuration. In our study, we considered grade A as an excellent/unimpaired graft, and grafts that showed type B or O were occluded.

### 2.4. Statistical analysis

Continuous variables were reported as standard deviation and categorical variables as frequency and proportion. Analysis between the patent and occluded groups was compared by unpaired *t* test for continuous variables and with chi-square test or Fisher’s exact test for categorical variables. Multivariate logistic regression analysis was performed to identify independent risk factors for 5-year graft failure. *P* < .05 were considered significant. SPSS statistic version 25 was used.

## 3. Results

Table 1 shows baseline characteristics. In the study cohort, no deaths in the hospital, all patients’ angina was relieved after surgery, and no remarkable complications were discharged. The 202 patients underwent primary isolated off-pump CABG at a mean age of 58.6 ± 7.3 years, 66 (32.7%) with diabetes and 136 (67.3%) without diabetes. Intraoperative graft means graft flow (MGF) was significantly lower in the diabetic group (27.1 ± 16.3) than that in the nondiabetic group (36.4 ± 24.1) (*P* = .001), and pulsatile index was significantly higher in the diabetic group (2.5 ± 0.9) than that in the nondiabetic group (2.2 ± 0.8) (*P* = .011). Furthermore, the proportions of female gender and hypertension were increased in the diabetic group.

**Table 1:** characteristics of the study cohort

Variable	Total (n = 202)	Diabetic (n = 66)	Nondiabetic (n = 136)	*P* value
Demographics				
Age (range)	58.6 ± 7.3 (38–76)	58.2 ± 7.5 (38–75)	58.8 ± 7.2 (42–76)	.630
Female (%)	21.3	30.3	16.9	.029
BMI (kg/m^2^)	25.8 ± 3.1 (15.4, 35.5)	25.9 ± 3.3 (15.4, 35.5)	25.8 ± 3.0 (18, 34.9)	.856
Cardiovascular risk factors				
Hypertension (%)	119 (58.9)	48 (72.7)	71 (52.2)	.005
Hyperlipidemia	55	23	32	.090
Current smoker (%)	118 (58.4)	37 (56.1)	81 (59.6)	.636
Stroke (%)	12 (5.9)	3 (4.5)	9 (6.6)	.754
Renal disease	5	2	3	.663
COPD	15	3	12	.394
Coronary lesion				
Single vessel disease	7	2	5	1
Two-vessel disease	43	13	30	.701
Three-vessel disease	152	49	103	.818
Angina class				
CCS I–II	190	60	130	.187
LVEDD (mm)	48.3 ± 4.8 (34, 60)	47.7 ± 5.1 (34, 60)	48.6 ± 4.7 (38, 60)	.228
LVEF (%)	61.8 ± 5.9 (50, 77)	62.7 ± 6.7 (50, 77)	61 ± 6.8 (50, 77)	.220
LVEF ≥55 (%)	179 (88.6)	62 (93.9)	117 (86)	.842
Euro SCORE	0.8 ± 0.8 (0, 4)	0.7 ± 0.8 (0, 3)	0.8 ± 0.9 (0, 4)	.947
Intraoperative TTFM				
MGF (mL/min)	33.4 ± 22.3 (6130)	27.1 ± 16.3 (6, 78)	36.4 ± 24.1 (6130)	.001
PI	2.3 ± 0.9 (1, 6)	2.5 ± 0.9 (1, 5)	2.2 ± 0.8 (1, 6)	.011
DF (%)	71.3 ± 8.3 (35, 99)	70.8 ± 9.1 (35, 95)	71.0 ± 8.2	.882

BMI = body mass index, CCS = Canadian Cardiovascular Society, COPD = chronic obstructive pulmonary disease, DF = diastolic filtration, LVEDD = left ventricular end-diastolic diameter, LVEF = left ventricular ejection fraction, MGF = mean graft flow, PI = pulsatile index, TTFM = transit-time flow measurement.

**Figure 2.**
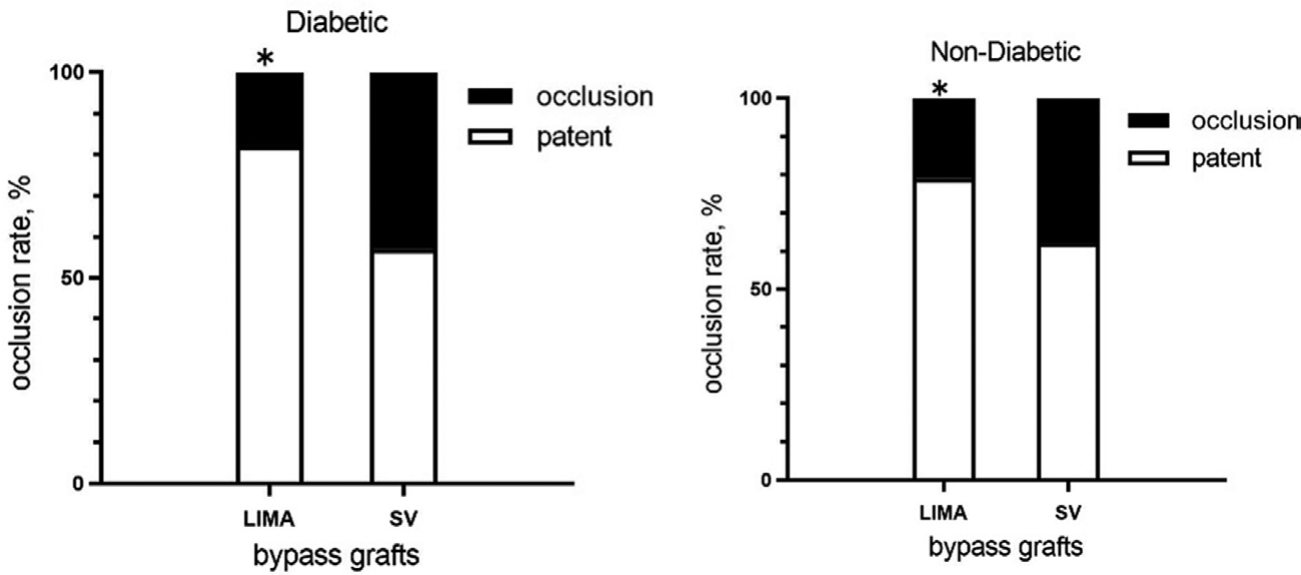
The 5-yr angiography follow-up of diabetic patients and nondiabetic patients. *P < .05. LIMA = left internal mammary artery, SV = saphenous vein.

In the diabetic group, the number of bypass grafts of LIMA and SV were 66 and 133, respectively. The 5-year postoperative angiographic showed the overall graft occlusion rates of LIMA and SV were 18.2% (54/66) and 42.9% (76/133), respectively. In the nondiabetic group, the number of bypass grafts of LIMA and SV were 136 and 275, respectively. The 5-year postoperative angiographic showed the overall graft occlusion rate of LIMA and SV were 20.6% (108/136) and 38.2% (170/275), respectively (Fig. 2).

**Figure 3.**
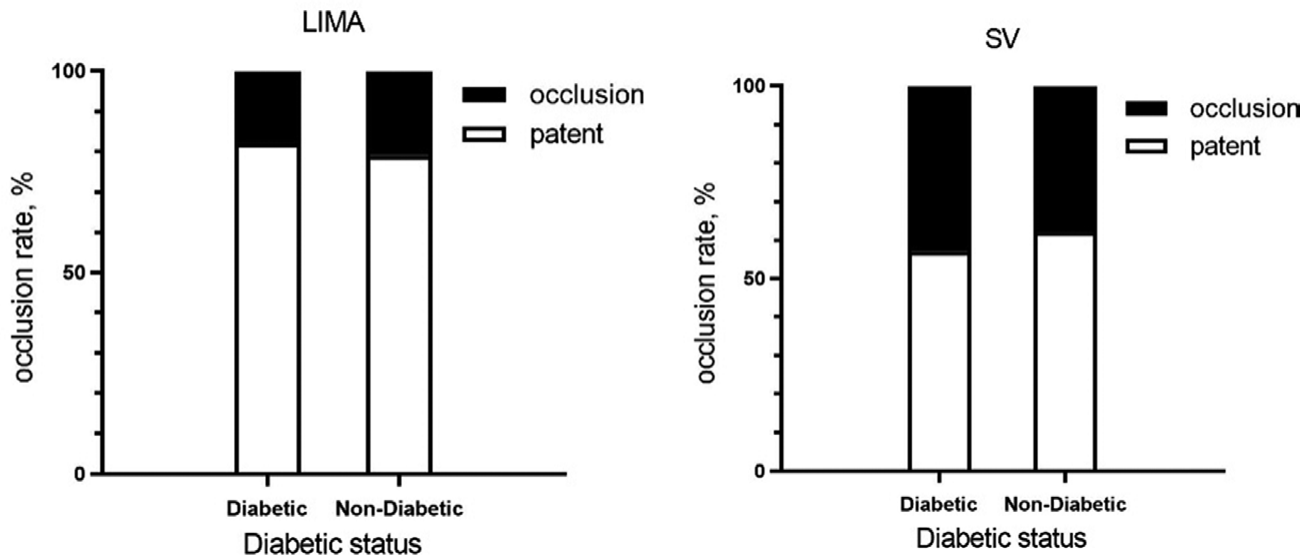
Comparison of complete graft occlusion of the same conduit between diabetic and nondiabetic patients. LIMA = left internal mammary artery, SV = saphenous vein.

Within the graft subtypes, the proportion of occluded LIMA grafts was similar irrespective of diabetic status (12/66, 18.2% in the diabetic group vs 28/136, 20.6% in the nondiabetic group, *P* = .687). The proportion of occluded SV grafts was also similar irrespective of diabetic status (57/133, 42.9% in diabetic group vs 105/275, 38.2% in nondiabetic group, *P* = .366) (Fig. 3).

Multivariate logistic regression analysis was performed to identify independent risk factors of 5-year graft failure (Table 2). MGF were the only significant predictor of LIMA graft failure (odds ratio = 1.186, 95% CI = 1.114–1.263, *P* < .001). MGF were also the only significant predictor of SV graft failure (odds ratio = 1.056, 95% CI = 1.035–1.077, *P* < .001).

**Table 2:** Multivariate predictors of graft failure

Variables	LIMA graft			SV graft		
	OR	95% CI	*P* value	OR	95% CI	*P* value
Female	0.543	0.136–2.175	.389	1.107	0.495–2.476	.804
Age	0.990	0.919–1.067	.796	1.010	0.974–1.047	.596
BMI	1.082	0.921–1.271	.337	1.016	0.948–1.08	.656
Smoking	1.881	0.617–5.736	.267	0.906	0.473–1.735	.767
Hypertension	1.253	0.459–3.424	.659	0.925	0.536–1.596	.779
Diabetes mellitus	2.644	0.957–7.306	.061	0.892	0.508–1.566	.691
Stroke	3.939	0.343–45.178	.271	2.093	0.705–6.217	.184
LVEF	0.970	0.906–1.038	.378	1.034	0.995–1.074	.089
MGF	1.186	1.114–1.263	<.001	1.056	1.035–1.077	<.001
PI	1.155	0.710–1.881	.562	0.909	0.742–1.113	.356
DF	1.005	0.947–1.068	.861	1.018	0.992–1.044	.170

BMI = body mass index, DF = diastolic filtration, LIMA = left internal mammary artery, LVEF = left ventricular ejection fraction, MGF = mean graft flow, PI = pulsatile index, SV = saphenous vein.

## 4. Discussion

In this study, we found that LIMA bypass grafts had higher patency rates at 5-year follow-up compared with SV bypass grafts of patients with or without diabetes. In addition, the principle finding of the present study is that diabetes did not influence the process of occlusion of bypass grafts. LIMA graft was superior to SV grafts on long-term graft patency.^[14]^ In our study, 5-year angiographic follow-up showed the overall graft patency rate of LIMA and SV were 80.2% (162/202) and 60.3% (246/408), respectively. LIMA grafts are resistant to atherosclerosis, and they are more likely to keep patents compared with SV grafts.^[15–18]^ However, SV grafts had the potential to decrease patency affected by the progression of atherosclerosis.^[19–21]^

Intraoperative graft assessment was recommended in previous guidelines.^[22,23]^ In these guidelines, MGF should be more than 20 mL/min and pulsatile index less than 5. In the present study, in LIMA grafts, MGF was significantly higher in patent grafts compared with occlusion grafts (38.1 ± 22.3 vs 14.2 ± 5.8(6,27), *P* < .001). In SV grafts, MGF was also significantly higher in patent grafts compared with occlusion grafts (36.3 ± 21 vs 18.8 ± 13.1, *P* < .001). Multivariate logistic regression analysis showed that MGF was only an independent predictor factor for graft failure.

There still exist debates about the effect of diabetes on bypass graft patency. The study^[8]^ found similar graft patency in patients with and without diabetes at 3.9-year angiographic follow-up. Internal thoracic artery graft patency was 89% in patients with diabetes versus 85% in patients without diabetes (*P* = .20), and SV graft patency was 71% versus 75% (*P* = .40), respectively. In addition, the study^[9]^ found similar graft patency in patients with and without diabetes at 5-year angiographic follow-up. On the contrary, the study^[10]^ found SV graft patency was 75% in patients with diabetes versus 84% in patients without diabetes (*P* = .06) by at least 5 years of angiography follow-up. Ayan et al^[11]^ found similar arterial graft patency in matched patients with and without diabetes, but worse SV graft patency in patients with diabetes.

In addition, many factors affect the patency of the grafts, including the quality, size, and diameter of the SV; the size and diameter of the target coronary artery; surgical skills; intraoperative handling of vein graft material; and (perioperative and postoperative) medical management. Most of all, the SV grafts may fail due to issues in handling the vein graft during the harvesting procedure and the harvesting process itself. In the study, all SVG was obtained with open SVG harvesting. With the improvements in method, other harvesting techniques including open, bride, no-touch, and endoscopic, were used for the SVG. A consensus regarding the best SVG harvesting technique has not yet been reached.^[23]^ Whether diabetes affects SVG differently depending on the method remains to be further investigated.

This study had some limitations. It was a retrospective study rather than a randomized study. In addition, this study had a relatively small sample size, and the off-pump CABG surgery was performed at a single center. Finally, the diagnosis of diabetes was based on clinical criteria at baseline and was not rigorously defined. We did not collect information about the long-term use of diabetes medications, and we did not have information on the degree of glycemic control, hypoglycemia rates, and lipid and blood pressure management.

## 5. Conclusions

Diabetes did not influence the 5-year patency of LIMA graft. The use of LIMA grafts should be maximized in patients undergoing off-pump CABG surgery. Cardiac surgeons should pay more attention to the intraoperative MGF. Totally, via angiography skills, we analyzed that diabetes does not affect the patency of grafted vessels after CABG for 5 years follow-up.

## Author contributions

**Data curation:** Caiwu Zeng, Xiaomi Li.

**Formal analysis:** Caiwu Zeng, Nan Liu.

**Writing – original draft:** Caiwu Zeng, Nan Liu.

**Writing – review & editing:** Caiwu Zeng.

**Conceptualization:** Xiaomi Li, Ye Zhou.

**Investigation:** Xiaomi Li, Ye Zhou, Nan Liu.

**Software:** Ye Zhou.
